# Flexible Inorganic/Organic Memristor Based on W-Doped MoO_x_/Poly(methyl methacrylate) Heterostructure

**DOI:** 10.3390/nano15221707

**Published:** 2025-11-12

**Authors:** Gion Kalemai, Konstantinos Aidinis, Elias Sakellis, Petros-Panagis Filippatos, Polychronis Tsipas, Dimitris Davazoglou, Anastasia Soultati

**Affiliations:** 1 Department of Physics, University of Patras, Rio, 26504 Patra, Greece; c.kalemai@iit.demokritos.gr; 2 Department of Electrical and Computer Engineering, Ajman University, Ajman P.O. Box 346, United Arab Emirates; 3 Center of Medical and Bio-Allied Health Sciences Research, Ajman P.O. Box 346, United Arab Emirates; 4 Institute of Nanoscience and Nanotechnology (INN), National Center for Scientific Research Demokritos, Agia Paraskevi, 15341 Athens, Greece; e.sakellis@inn.demokritos.gr (E.S.); petpanfilippatos@gmail.com (P.-P.F.);; 5 Solid State Physics Section, Department of Physics, National and Kapodistrian University of Athens, Panepistimioupolis, Zografos, 15784 Athens, Greece

**Keywords:** molybdenum oxide, doping, tungsten, PMMA, resistive switching, heterostructire, memristor

## Abstract

Work investigates the doping of molybdenum oxide (MoO_x_) with tungsten (W). The successful incorporation of W into the MoO_x_ lattice was confirmed through X-ray photoelectron spectroscopy (XPS) and energy-dispersive X-ray spectroscopy (EDS). Structural and optical analysis revealed the presence of oxygen vacancies within the W-MoO_x_ film, which are known to facilitate resistive switching (RS) in memristive devices. Based on this, a flexible memristor with the structure PET/ITO/W-MoO_x_/polymethyl methacrylate (PMMA)/Al was fabricated. PMMA was strategically introduced between the W-MoO_x_ layer and the aluminum electrode to modulate interfacial properties that influence RS behavior. The W-MoO_x_/PMMA-based memristor exhibited good resistive switching characteristics, with a memory window of approximately 12 and a retention time exceeding 2 × 10^4^ s, demonstrating a non-volatile memory behavior. In the high-resistance state (HRS), the conduction mechanism under higher applied voltages follows a space-charge-limited current (SCLC) model, indicating that the RS process is primarily governed by charge trapping and de-trapping at the interface. Overall, the consistent and robust switching performance of the W-MoO_x_/PMMA heterostructure underlines its potential as a reliable functional layer for next-generation resistive random-access memory (ReRAM) devices.

## 1. Introduction

In modern life, the growing demand for data processing and storage has put great pressure on computing hardware. From smartphones to cloud-based systems, large amounts of data are generated and analyzed every day. This need calls for more powerful and efficient computing solutions. As a result, artificial intelligent devices have taken on an important role, especially in designing and optimizing neural network configurations [1,2,3]. These systems inspired by the human brain need a lot of processing power and storage to function effectively. Consequently, much effort has been made in developing high-density information storage technologies. Memristor-based resistive random-access memory (ReRAM) is a promising candidate for next-generation computing architectures, due to the ease of the device structure and monolithic integration, rapid switching speed, and low power consumption [4,5]. Resistive switching (RS) devices using various materials as functional or active layers have been reported, including metal oxides [6,7,8], transition metal dichalcogenides [9,10,11], organic semiconductors [12,13], and organic–inorganic halide perovskites [14,15,16].

Transition metal oxides (TMOs) are a class of materials widely used in memristors. Molybdenum oxide (MoO_3_) and tungsten oxide (WO_3_) are two TMOs that have received significant attention for their unique properties including wide optical bandgap, eco-friendly nature, and excellent electrical and thermal stability, which make them ideal materials for memristive devices [17,18]. Particularly, MoO_3_’s high stability under different conditions, along with its ability to store and release charge efficiently, makes it a reliable material for resistive switching applications [19]. MoO_3_ also demonstrates artificial synaptic properties, which are important for neuromorphic computing imitating synaptic behavior [20]. Moreover, WO_3_ exhibits good stability and a high capacity for charge storage, making it a strong candidate for memristors, especially in applications that need high endurance and reliability. More importantly, the electrical properties of both MoO_3_ and WO_3_ can be adjusted by modifying their stoichiometry, allowing greater control over the switching characteristics and performance of memristive devices [21,22]. Their resistive switching behavior is closely related to oxygen vacancies within the oxide. The distribution and density of these vacancies greatly influence the conductive mechanisms that drive the switching process. Therefore, controlling the oxygen content within the resistive switching layer is essential for achieving the desired performance in memristors.

Doping in metal oxide-based memristors is an efficient approach for improving the electrical performance and reliability of the devices. By adding dopants like aluminum (Al) [23], lithium (Li) [24], or nickel (Ni) [25] into the metal lattice, the oxygen vacancy concentration, electronic conductivity, and switching thresholds can be tuned. Moreover, Li doping boosts ionic mobility, allowing for faster switching and improved synaptic behavior in neuromorphic applications [26]. Doping of molybdenum oxide-based memristors has also been investigated. In particular, silver (Ag) [27] and copper (Cu) [28] doping of MoO_x_ resulted in controlled oxygen vacancies, which led to better switching uniformity and endurance. These dopants tune the local electronic structure and defect concentration, influencing filament formation, charge trapping, and overall device stability. Consequently, doped MoO_x_ memristors showed higher ON/OFF ratios, lower variability, and longer retention times.

In this work, the doping of molybdenum oxide (MoO_x_) with tungsten (W) is investigated, motivated by the complementary and favorable electronic properties of both MoO_3_ and WO_3_. The fabrication of a W-doped MoO_x_-based flexible memristor lies in the unique potential of tungsten doping to modulate the electronic structure and oxygen vacancy dynamics of MoO_x_, thereby influencing switching behavior. While memristors based on MoO_x_ have been studied previously, W-doping remains relatively underexplored in flexible devices. Our work focuses on the trade-offs involved in developing flexible devices, where performance is often constrained by mechanical and thermal limitations. In our approach, the W-MoO_x_ film is formed using hot-wire chemical vapor deposition, where the substrate remains at room temperature and no further annealing process is performed. Consequently, this study offers valuable experimental insights into the feasibility, stability, and resistive switching characteristics of W-doped MoO_x_ devices implemented on flexible substrates. X-ray photoelectron spectroscopy (XPS) and energy dispersive X-ray spectroscopy (EDS) confirmed the presence of W in the MoO_x_ lattice. The structural, optical, and electronic properties of the deposited W-MoO_x_ were also investigated using various technique characterization methods including X-ray diffraction (XRD), Fourier transform infrared spectroscopy (FTIR), ultraviolet–visible (UV-vis) spectroscopy, photoluminescence spectroscopy (PL), and ultraviolet photoelectron spectroscopy (UPS). It was found that oxygen vacancies existed in the developed W-MoO_x_ film, which could facilitate the resistive switching (RS) of a memristor. Therefore, a flexible memristive device with the PET/ITO/W-MoO_x_/polymethyl methacrylate (PMMA)/Al structure was fabricated. The PMMA was inserted between the W-doped molybdenum oxide and the aluminum in order to control interfacial properties influencing the RS behavior of the device. The memristor based on the W-MoO_x_/PMMA heterostructure exhibits stable resistive switching with a memory window of approximately 12 and a retention time of 2 × 10^4^ s, confirming its non-volatile behavior. In the high-resistance state (HRS) under high applied voltages, the conduction mechanism follows space-charge-limited current (SCLC), indicating that the resistive switching is primarily governed by an interface-driven mechanism involving charge trapping and de-trapping. The consistent switching performance highlights the effectiveness of the W-MoO_x_/PMMA heterostructure as a functional layer for reliable ReRAM applications.

## 2. Experimental Section

### 2.1. Film Preparation and Characterization

A tungsten (W)-doped molybdenum oxide (named hereafter as W-MoO_x_) thin film was deposited on various substrates including poly(ethylene terephthalate) (PET) coated with indium-tin oxide (ITO), glass, and silicon (Si) using a chemical vapor deposition (CVD) method. In particular, a hot-wire CVD system consisting of a stainless steel reactor, an aluminum base, and copper leads was used for the development of W-MoO_x_ thin films. Between the two copper leads, two wires, one molybdenum and one tungsten, with a diameter of 0.5 mm were placed to form the W-doped molybdenum oxide. The deposition process started with the evacuation of the reactor chamber to a base pressure of 10 mTorr using a mechanical pump. W-MoO_x_ films were obtained using forming gas, a mixture consisting of 90% nitrogen and 10% hydrogen. Films were deposited at a chamber pressure of 80 mTorr, with a molybdenum and a tungsten wire heated to 560 °C serving as the evaporation source. The gas flow rate was maintained at 100 ppm throughout the process. The deposition duration was 90 s, resulting in film thicknesses of approximately 100 nm.

Elemental composition identification was conducted using scanning electron microscopy (SEM) provided by Thermo Fisher Scientific Inc., Waltham, MA, USA. Particularly, a variable pressure FEI Quanta microscope (FEI Company, Hillsboro, OR, USA) equipped with an EDAX Energy Dispersive X-ray Spectroscopy (EDS) detector (AMETEK, Inc., Berwyn, PA, USA) was used. X-ray photoelectron spectroscopy (XPS) was performed using a Mg Kα radiation source (photon energy: 1253.64 eV) to probe the elemental composition and chemical states at the sample surface. The spectra were acquired with a PHOIBOS 100 mm hemispherical analyzer (SPECS GmbH, Berlin, Germany), operated at a pass energy of 7 eV to achieve high energy resolution. All measurements were conducted under ultra-high vacuum conditions, with base pressures maintained below the 10^−8^ mbar range. For ultraviolet photoelectron spectroscopy (UPS), a He I (21.22 eV) discharge lamp was used as the excitation source to investigate the valence band structure and the work function of the sample. The same hemispherical analyzer was employed to collect the emitted photoelectrons. The structural analysis of the W-MoO_x_ films was investigated using a Rigaku Smart Lab X-ray Diffractometer (Rigaku SmartLab, Neu-Isenburg, Germany) with Cu-Ka radiation. Θ/2Θ scans were performed, and the angular range for data collection was 2.0–80.0°, scanned in steps of 0.03° with a scan speed of 0.3 s/step. The surface nanomorphology of the W-doped molybdenum oxide films was studied with an NT-MDT AFM system (LaborScience SA, Athens, Greece) operating in tapping mode. Photoluminescence measurements were performed using a Shimadzu RF-6000 spectrofluorophotometer (Asteriadis, Thessaloniki, Greece). The thickness and the reflectance of the prepared W-MoO_x_ films were measured using an FR-pRo UV/NIR-HR (SPS Polos, Putten, The Netherlands) operating in the 190–1100 nm spectral range, capable of measuring film thickness in the 1 nm–100 μm spectral range. The UV–visible absorbance and transmittance spectra of the W-doped molybdenum oxide were recorded with a Shimadzu UV-1900-i spectrometer (Asteriadis, Greece). Fourier transform infrared spectroscopy was performed using a Fourier Bruker Tensor 27 spectrometer (Bruker, Billerica, MA, USA) (at 4 cm^−1^ resolution, 64 scans) equipped with a DTGS detector. Cyclic voltammetry measurements were obtained using a VersaSTAT4 potentiostat using an Ag/AgCl electrode as a reference.

### 2.2. Memristive Device Fabrication and Characsterization

Memristors were fabricated following the structure PET/ITO/W-MoO_x_/poly(methyl methacrylate) (PMMA)/Al. First, the PET/ITO substrates, which were used as the bottom electrode of the memristor, were subjected to a cleaning routine, where the substrates were placed in an ultrasonic bath of deionized water, acetone, and isopropanol for 10 min each. Then, the substrates were dried by a flow of nitrogen and placed in the hot-wire CVD system. The W-doped MoO_x_ oxide was deposited on the PET/ITO, forming a 100 nm thick film. Next, a PMMA layer (~80 nm) was spin-coated on the mixed oxide film from a methyl isobutyl ketone solution with a concentration of 1 mg mL^−1^. The device was completed with the thermal deposition of an aluminum layer, which served as the top electrode of the memristor.

The electrical measurements of the fabricated memristor were conducted using a VersaSTAT4 potentiostat. The bottom-ITO and the top-Al electrodes were connected to the ground and bias, respectively.

## 3. Results and Discussion

### 3.1. Deposition of W-Doped MoO_x_ Thin Films

**Figure 1:**
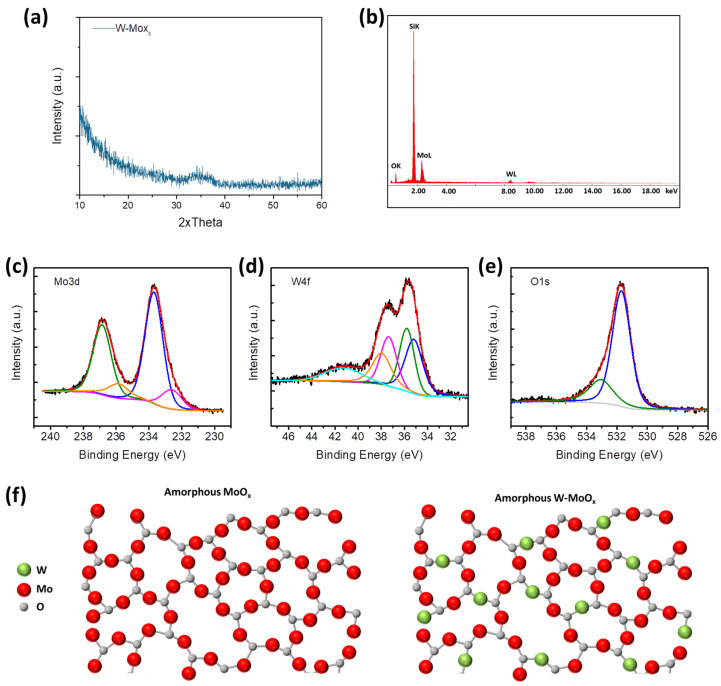
(**a**) XRD patterns and (**b**) EDS image of W-doped MoO_x_ film deposited on silicon substrate. XPS (**c**) Mo 3d, (**d**) W 4F, and (**e**) O 1s of W-MoO_x_ film. (**f**) Schematic illustration of the substitution of Mo atoms with Mo atoms forming the W-MoO_x_ film.

Tungsten (W)-doped molybdenum oxide films of 100 nm thickness were prepared using a hot-wire CVD reactor. Two wires, one of tungsten and another of molybdenum, were used for the deposition of the W-MoO_x_ films. During the deposition, a reduced gas consisting of 90% nitrogen (N_2_) and 10% hydrogen (H_2_) was flown into the CVD reactor to form a sub-stoichiometric W-doped molybdenum oxide, as described in previous work [29,30]. The structural properties of the W-MoO_x_ mixed oxide were firstly studied. Figure 1a shows the X-ray diffraction (XRD) pattern of the doped oxide film, where any diffraction peak appeared, leading to the conclusion that the W-MoO_x_ film is amorphous. The elemental analysis of the developed oxide film was performed using energy dispersive X-ray spectroscopy (EDS). Figure 1b shows the EDS image of the W-MoO_x_, while the quantitative elemental analysis is summarized in Table 1. The W-doping of molybdenum oxide was successfully achieved due to the substitution of Mo atoms with W atoms during the CVD procedure. The remarkable similarities between the molybdenum and tungsten ionic radii (0.059 nm and 0.06 nm for Mo and W, respectively), valence band, and electronegativity facilitated the incorporation of W into the MoO_x_ lattice [31]. In our CVD system, the molybdenum (Mo) and tungsten (W) wires are co-located within the deposition chamber, which limits the ability to precisely control or vary the W-doping concentration during film growth. The final doping level is inherently determined by the fixed relative positioning and evaporation characteristics of the precursor materials, i.e., the two wires. Consequently, independent tuning of the W content is not feasible in the current setup. To address this constraint, the study focused on optimizing other process parameters, particularly film thickness, to ensure consistent and reliable device performance. Another approach to controlling the concentration of doping is by changing the environment of deposition. The deposition of MoO_3-x_ in a nitrogen (N_2_) environment decreased the concentration of doping as shown in the EDS image in Figure S1 and Table S1. The devices investigated in this work incorporate a W atomic ratio of 9.72%, representing an optimized configuration identified through extensive preliminary experimentation. Although detailed data from the optimization studies (e.g., film thickness variations, environment of deposition) are not presented here, they guided the selection of this specific composition. The aim of this work is to study and demonstrate resistive switching behavior in flexible W-doped MoO_x_ devices, for which the chosen doping level was found to be effective. The surface elementary composition of the W-doped molybdenum oxide was also investigated by X-ray photoelectron spectroscopy (XPS). Figure 1c presents the XPS Mo 3d peak, which appears as a doublet due to spin–orbit splitting. The peaks with binding energy (BE) at 236.7 eV and 233.6 eV correspond to the higher oxidation state (Mo^6+^) of Mo cations, while the peaks at 235.9 eV and 232.7 eV BE are assigned to the Mo^5+^, confirming the sub-stoichiometric nature of the molybdenum oxide sample [32,33]. Apart from the Mo^6+^ and Mo^5+^, the appearances of W^6+^ at 37.4 eV and 35.8 eV BE and of W^5+^ located at 38.0 eV and 35.2 eV BE are observed in the XPS spectrum of the sample (Figure 1d showing the W 4f XPS spectrum), indicating the deposition of a sub-stoichiometric W-doped molybdenum oxide film [34,35]. Moreover, the peak at 533 eV BE in the O 1s XPS spectrum of the sample is assigned to the hydroxyl groups incorporated in the W-MoO_x_ lattice during the deposition using a reduced environment (90%/10% N_2_/H_2_). Figure 1f illustrates the development of amorphous W-doped MoO_x_, showing the substitution of Mo cations with W^6+^ and W^5+^ in the amorphous MoO_x_ lattice. In addition, the reduced deposition environment and the low deposition pressure of ~80 mTorr reduced the evaporation rate of MoO_x_, which favors the W-doping process [36].

**Figure 2:**
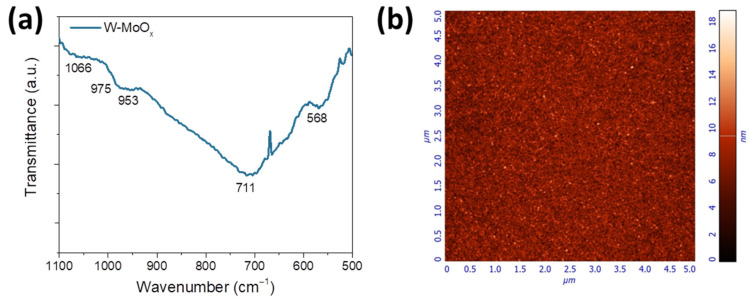
(**a**) FTIR transmittance spectrum of W-MoO_x_ thin film. (**b**) 5 × 5 μm^2^ AFM images of the surface of the same sample.

The structure of the W-MoO_x_ film was also investigated using FTIR spectroscopy. Figure 2a presents the FTIR transmittance spectrum of the W-doped oxide film. In the molybdenum oxide FTIR spectrum shown in Figure S2a (Supporting Information), a peak at about 713 cm^−1^ appears, which is related to the bending vibration of the Mo–O bond. Moreover, the peak at 984 cm^−1^ is assigned to the stretching mode of the Mo=O bond, while the weak peak at around 848 corresponds to the stretching vibration of the Mo–O bond. The new peaks appearing in the FTIR spectrum of the W-doped MoO_x_ at about 975 cm^−1^ and 953 cm^−1^ could be a combination of the vibrations of Mo=O and W=O. The peak at 568 cm^−1^ is related to the stretching mode of the Mo–O bond. Another broad peak at 568 cm^−1^ also appears in the W-MoO_x_ FTIR spectrum, which could be attributed to the shift of the W–O band (Figure S2b) to lower wavenumbers (from 625 cm^−1^ to 568 cm^−1^). Furthermore, the peak at around 1066 cm^−1^ is assigned to the bending mode Mo–OH and W–OH bonds formed during the deposition of the W-MoO_x_ oxide in a reduced environment [37].

In order to investigate the nanomorphology of the W-doped MoO_x_ film, atomic force microscopy (AFM) measurements were performed. Figure 2b shows the 5 × 5 μm^2^ AFM 2D height image. It is observed that the surface of the mixed oxide film is smooth, exhibiting a root mean square (RMS) roughness of 1.8 nm. Moreover, the W-MoO_x_ surface consists of small grains in a range of 20–36 nm, as revealed by the grain analysis presented in Figure S3, which was performed in the 5 × 5 μm^2^ AFM image.

**Figure 3:**
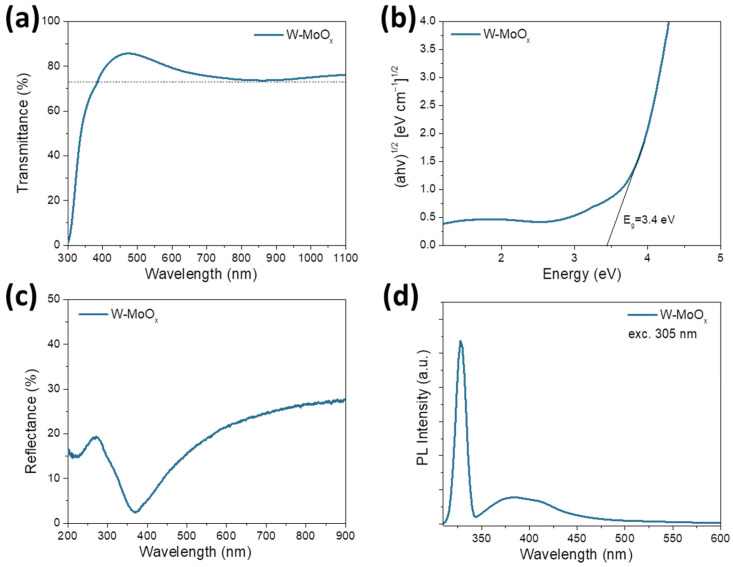
(**a**) Transmittance spectrum, (**b**) Tauc plot, (**c**) reflectance spectrum, and (**d**) photoluminescence spectrum of W-doped MoO_x_ deposited on glass substrate.

The optical properties of the developed W-MoO_x_ sample were also studied using ultraviolet–visible (UV-Vis) spectroscopy. The prepared W-doped oxide film deposited on the glass substrate exhibited high transparency, over 73%, in the visible region, as shown in the transmittance spectrum in Figure 3a. The optical bandgap (E_g_) of the W-MoO_x_ film was also estimated using the absorption measurements presented in Figure S4. As revealed from the corresponding Tauc plot ((ahv)^1/2^ versus energy, where a is the absorption coefficient and hv is the photon energy) shown in Figure 3b, the W-MoO_x_ sample exhibited an E_g_ of approximately 3.4 eV. Figure 3c shows the reflectance of the W-doped molybdenum oxide film, where the signal response in the UV region (200–400 nm) is assigned to the optical bandgap of the W-MoO_x_ (3.4 eV). On the other hand, in the visible region, the W-doped oxide exhibited a broad absorption which could be related to the concentration of oxygen vacancies increasing the reflectivity of the film [38]. The oxygen vacancies in the W-MoO_x_ are also evident in the photoluminescence (PL) spectrum of the film (Figure 3d). In particular, the broad peak in the visible region (~384 nm) could be assigned to d-d band transitions, while the weaker peak at around ~414 nm could be attributed to the existence of defect and oxygen vacancies of the mixed oxide. Note that the peak at 328 nm located close to the near band edge (NBE) emission is mainly due to the free exciton recombination. The existence and concentration of oxygen vacancies may facilitate the resistive switching mechanism in a memristor.

### 3.2. Computational Results

To confirm our experimental findings, we performed density functional theory (DFT) calculations for the perfect MoO_3_ structure and the defected-with-W structure. Our calculations were carried out using the Vienna Ab initio Simulation Package (VASP) [39,40,41]. We employed spin-polarized DFT with projector augment wave (PAW) pseudopotentials [39] for all species. The exchange-correlation effects were described by the regularized-restored strongly constrained and appropriately normed (r_2_SCAN) meta-GGA functional [42] with DFT-D3 dispersion corrections [43] to improve the accuracy of the lattice parameters, which were converged within 1.50% of the experimental values.

To ensure the accuracy of our results, we performed convergence tests for the determination of cut-off energy and k-point mesh. A plane-wave basis set with an energy cut-off of 750 eV was used in combination with a 4 × 4 × 2 k-point sampling grid. We chose these values after following the criteria of 1 meV/atom convergence of total energy for one electronic self-consistent field (SCF) iteration. The residual forces on all atoms were below 0.01 eV/Å. To minimize interactions between periodic image of defects, we used a large supercell of 128 atoms which had at least 10 Å in every direction. To find the correct ground state as well as the formation energy, we used the packages ShakeNBreak and Doped [44,45].

To predict the formation energy as a function of the fermi level, we followed the methodology that is described in previous work [46]. Through this approach, we can identify the stable charges of the defect as well as the charge transition levels. For a defect D at a charge state q, the formation energy is calculated from

(1) Eform[Ef,μ]=Etot[Dq]−EH−∑niiμi+qEf+Ecorr

where *E_tot_* [*D^q^*] is the total energy of the defect *D* at charge *q* and *E_H_* is the energy of the defect-free supercell of MoO_3_. *ni* represents the number of atoms that are added or removed with chemical potentials *μi*. The chemical potentials are variables that depend on the experimental synthesis. In this work, *μ_O_* is the total energy of a single atom in the O_2_ molecule, *μ_Mo_* is the energy of a single atom in the bulk Mo crystal, and *μ_W_* is the energy of a W atom in the bulk W crystal.

The perfect crystalline MoO_3_ is a van der Waals structure that belongs to the space group *Pnma*. Each structural layer consists of two sublayers of distorted MoO_6_ octahedra which connect through edge sharing along the c-axis and corner sharing along the a-axis. In the stoichiometric form, MoO_3_ is a wide-bandgap material with an approximately 3.0 eV gap and a 4d^0^ electron configuration. The application of the r_2_SCAN functional for this material has never been reported before. Using this functional, we calculate all the optimized lattice parameters being within 1.50% error from the experimental value. Specifically, the experimental values that are reported for the *Pbnm* space group are 9 a = 3.96 Å, b = 13.85 Å, and c = 3.70 Å, while our r_2_SCAN+D3 values which are for *Pnma* space group are a = 3.68 Å, b = 3.90 Å, and c = 13.77 Å. These two groups have the exact same symmetry with the axis defined differently, so we expect to have the same properties [47].

Our calculated bandgap is at a value of 2.30 eV, which is underestimated compared to the experimental bandgap but is still improved compared to the Perdew–Burke–Ernzerhof (PBE) functional value of 1.95 eV [48]. As shown in previous studies, while meta-GGA tends to underestimate the bandgap, this does not affect the calculation and prediction of the stable charges or their optical transition properties and is a good alternative to the computationally expensive hybrid functionals [49,50,51].

To predict the stable charge states of the W_Mo_ in MoO_3_, we use the defect formation energy diagrams as described in the Methods above. For every case, we verify the spin multiplicity for each charge state to make sure that we predict the correct ground state for each charge state. Figure S5 shows the formation energy diagram for W_Mo_, where it is seen that, as a defect, it is stable in neutral and −1 charge. Our calculations represent the O-rich conditions. We find a low formation energy for the neutral defect at 0.22 eV. This result indicates that W on the Mo site should be energetically accessible under these conditions.

After identifying the stable charges and the formation energies, we now discuss the electronic structure of the stable configurations. In Figure S6a,b, we show the density of states for the neutral and −1 charge, respectively, as well as the undoped MoO_3_ (Figure S6c). From our analysis, we found that, in both charges, no gap states are created, so we expect that the peaks that are presented in the PL plot should be transitions from the VBM to the CBM. Furthermore, we find that the bandgap is slightly decreased with the W_Mo_ incorporation. The E_g_ of MoO_x_ with the same thickness was estimated for comparison reasons and is presented in Figure S6d.

### 3.3. Fabrication of Memristor Using W-MoO_x_/PMMA Heterostructure

**Figure 4:**
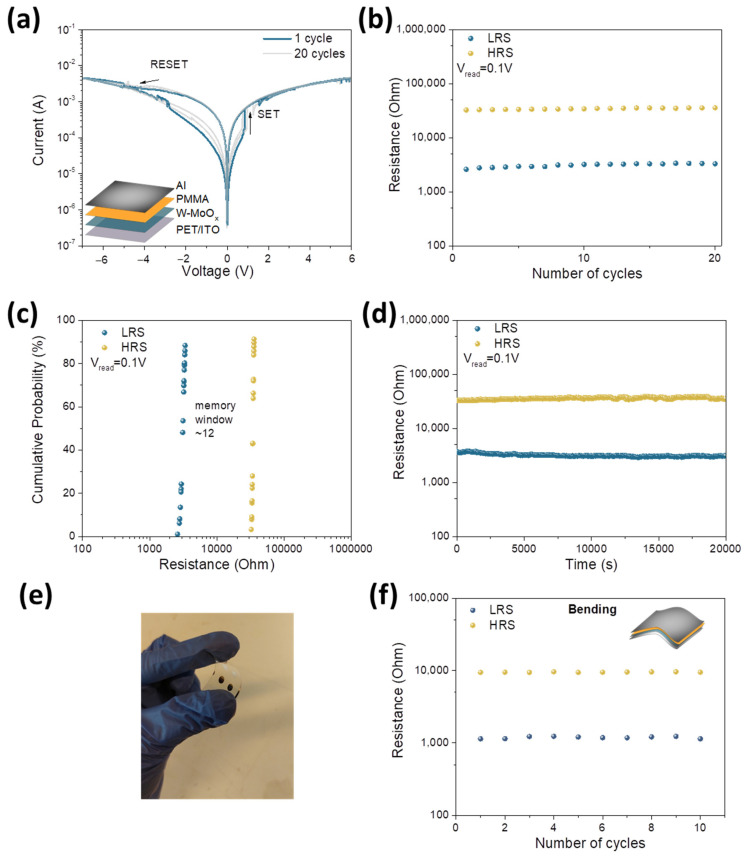
(**a**) I-V switching curves of the memristor based on W-MoO_x_ for 20 consecutive cycles of sweep voltages. The device structure is presented as an inset. (**b**) Endurance characteristics of the same memristor under 20 SET/RESET switching cycles. (**c**) Cumulative probability plots of HRS and LRS of the W-MoO_x_ memristor. (**d**) Retention performance of the LRS and HRS of the same device after SET and RESET operation. The pulse SET and RESET voltages were 1.2 V and −4 V, respectively, while the V_read_ was 0.1 V. (**e**) Photograph of flexible W-MoO_x_/PMMA-based memristor. (**f**) Endurance characteristic of the same device upon mechanical stress.

In order to investigate the resistive switching behavior of the W-doped molybdenum oxide, a flexible memristor based on the structure PET/ITO/W-MoO_x_/PMMA/Al was fabricated. A PMMA layer of 50 nm thickness spin-coated on the W-doped molybdenum oxide was used as a interfacial layer between the W-MoO_x_ and the Al. The prepared device, the schematic illustration of which is presented as an inset in Figure 4a, was electrically characterized to study its memristive performance. The resistive switching mechanism of the fabricated device is clearly evidenced by the hysteresis loop observed in the characteristic semilog current–voltage (I-V) curve shown in Figure 4a. The pristine state of the W-MoO_x_/PMMA device is the high-resistance state (HRS). The device transition from the HRS to the low-resistance state (LRS) resulting in the SET operation of the memristor is obtained by the application of positive voltage to the top Al electrode. This abrupt change in current occurred at around 0.8 V, which is the V_SET_ of the device. When a negative voltage is applied, the memristor gradually returns from the LRS to the HRS, leading to the RESET operation of the device. The resistive switching voltage at the RESET operation was around 3.1 V (V_RESET_).

An endurance test was also performed on the memristor based on the W-MoO_x_/PMMA heterostructure, recording the I-V characteristic curves over several cycles (Figure 4a). The memristor exhibited good endurance, maintaining the RS behavior for 20 cycles, as presented in Figure 4b. Moreover, the memory window of the device as estimated from the cumulative probability presented in Figure 4c was about 12, which is appropriate for ReRAM devices. A reference memristor without the PMMA layer was also fabricated for comparison reasons. Figure S7a,b show the I-V characteristic curves and the endurance of the device. It is observed that the memory window (~1.5) after 10 cycles is far smaller for bare W-MoO_x_ film compared with that of the heterostructure, indicating that the RS behavior is strongly correlated to the incorporation of the PMMA in the device. Figure 4d shows the retention performance of the W-MoO_x_/PMMA-based memristor, where, in the case of SET and RESET operation, a pulse with amplitude +1.2 V and −4V, respectively, was applied to the device. In addition, the reading voltage was set at 0.1 V. It is observed that the fabricated memristor retained the data over 2 × 10^4^ s, with no changes in the memory window between the LRS and HRS. The wide bandgap of the PMMA may limit the movement of trapped charge carriers after disconnecting the applied bias, helping to preserve the memristor’s state and leading to long retention times. The memristive behavior upon mechanical bending stress was also investigated. Figure 4e shows a photograph of our flexible memristor, and Figure 4f presents the endurance of the device in the bending state. The radius for the bending deformation was 5 mm. The LRS and HRS of the W-MoO_x_/PMMA memristor remained stable under mechanical stress, demonstrating the device’s potential for use in flexible memory applications.

**Figure 5:**
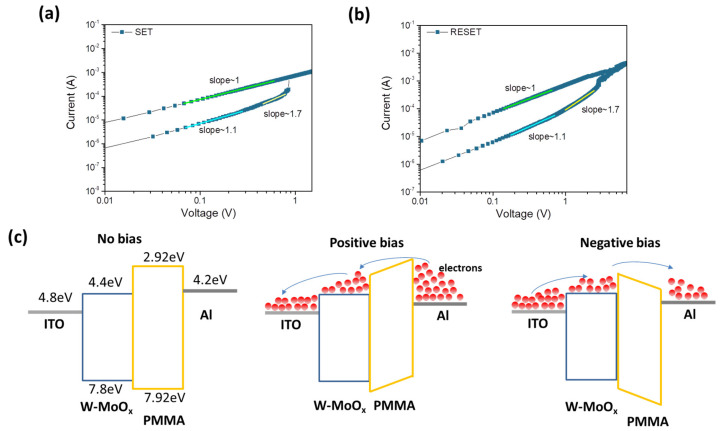
I-V curve analysis for conductive mechanism of the W-MoO_x_/PMMA-based memristor during (**a**) SET and (**b**) RESET operation. (**c**) Schematic illustration of the conductive mechanism of the ITO/W-MoO_x_/PMMA/Al device without bias application (**left**), with positive bias (**middle**), and with negative bias (**right**).

In order to investigate the conductive mechanism of the W-MoO_x_/PMMA-based memristor, the I-V characteristic curves of the SET and RESET operation were plotted on a double logarithmic coordinate axis, as depicted in Figure 5a and Figure 5b, respectively. At low voltages, in both cases (SET and RESET), the slope of the I-V is around 1, suggesting that the ohmic transition mechanism dominates, while in the region of high voltages, the slope of the fitting I-V curves is >1, indicating that the RS mechanism obeys the space charge limited conduction (SCLC) model. In order to shed light on the SCLC mechanism, the trap density (N_t_) was estimated using Mott–Gurney analysis (Equation (2)).

(2) $$V_{TFL}=\frac{qN_{t}d^{2}}{2\varepsilon_{ο}\varepsilon_{r}}$$

where V_TFL_ refers to the trap-filled limit voltage, q is the elementary charge, N_t_ refers to the trap density, d is the film thickness, and ε_ο_ and ε_r_ are the vacuum and relative permittivity, respectively. Based on Figure 5a,b, the trap density for the SET and RESET operation (assuming ε_r_ of ~3) [52] is ~1.66 × 10^16^ cm^−3^ and ~3.32 × 10^16^ cm^−3^, respectively. These values are in good agreement with previously reported trap densities [53] for oxide-based memristors, further validating the SCLC model in explaining the conduction mechanism of the W-doped MoO_x_ devices.

**Figure 6:**
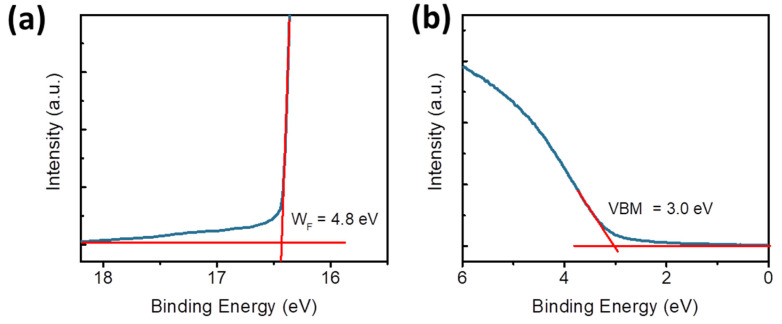
(**a**) High-energy cut-offs and (**b**) Fermi level regions of W-MoO_x_’s UPS spectrum.

Figure 5c illustrates the conductive mechanism of the ITO/W-MoO_x_/PMMA/Al memristor, where the left panel shows the energy level diagram of the memristor without bias. The energy levels presented in Note S1 of the W-doped MoO_x_ and PMMA films were estimated by cyclic voltammetry measurements, shown in Figure S8a,b, respectively [54]. The valence band (VB) of the W-MoO_x_ sample was also estimated using ultraviolet photoelectron spectroscopy. Figure 6a,b show the high-energy cut-off and Fermi level regions of W-MoO_x_’s UPS spectrum, respectively. The VB of the doped oxide is located 3 eV below the Fermi level and thus is estimated at −7.8 eV, in accordance with the cyclic voltammetry measurement, while the work function (W_F_) is 4.8 eV. When a positive voltage is applied to the top electrode (middle panel of Figure 5c), energy level bending occurs and the electrons reach the W-MoO_x_ layer, passing through the PMMA film. The electrons fill the traps in the W-MoO_x_ layer attributed to the oxygen vacancies of the W-doped molybdenum oxide film, as revealed by the reflectance and PL measurement, and when the filling is completed (at V_SET_), the conductive filament is formed. Therefore, the memristor switches from the HRS to LRS. On the other hand, when a negative voltage is applied to the Al, the electrons move reversely, leading to the depletion of the W-MoO_x_, the disruption of the conductive filament, and thus the LRS-to-HRS transition.

## 4. Conclusions

An inorganic/organic heterostructure consisting of W-doped molybdenum oxide and PMMA was used as functional layer in a flexible memristive device. The W-MoO_x_ film was developed using a hot-wire CVD method using Mo and W wires as the precursor materials. EDS and XPS analysis confirmed the presence of W in the MoO_x_ film, while the amorphous nature of the deposited W-MoO_x_ was evident from XRD measurements. The memristor based on the W-MoO_x_/PMMA heterostructure exhibited good resistive switching behavior, with a memory window of about 12 and retention time of 2 × 10^4^ s, indicative of the non-volatile behavior. In the case of HRS and at high applied voltages, the conductive mechanism obeys SCLC, suggesting that the memristor resistive switching behavior is an interface-dominated conductive mechanism attributed to the charge trapping and de-trapping effect. The good reproducibility of the memristor demonstrates the effectiveness of the W-MoO_x_/PMMA heterostructure as the functional layer in ReRAM devices.

## Figures and Tables

**Table 1:** Quantitative analysis, weight, and atomic ratios in percentage for oxygen (O, K series), molybdenum (Mo, L series), and tungsten (W, L series) of W-doped MoO_x_ sample.

Element	Weight Ratio (%)	Atomic Ratio (%)
O (K series)	21.76	68.08
Mo (L series)	42.55	22.20
W (L series)	35.69	9.72
Total	100	100

## Data Availability

The original contributions presented in this study are included in the article/Supplementary Materials. Further inquiries can be directed to the corresponding author.

## Supplementary Materials

The following supporting information can be downloaded at: https://www.mdpi.com/article/10.3390/nano15221707/s1. Figure S1: EDS image of W-doped MoO_x_ film deposited on silicon substrate in N_2_ environment; Figure S2: FTIR transmittance spectra of (a) molybdenum and (b) tungsten oxide thin films; Figure S3: Grain analysis of 5 × 5 μm^2^ AFM image of W-MoOx surface; Figure S4: Absorbance spectrum of the mixed W-MoO_x_ film deposited on glass substrate; Figure S5: The formation energy versus the fermi level for the O-rich conditions using r2SCAN+D3 functional; Figure S6: The density of states plot for the (a) neutral and (b) −1 charge W_Mo_^0^:MoO_3_ and (c) undoped MoO_3_; Figure S7: I-V characteristic curves of the PET/ITO/W-MoOx/Al memristor without the PMMA film; Figure S8: Cyclic voltammetry measurements of (a) W-MoOx and (b) PMMA films coated on ITO substrates; Note S1: Energy levels of W-MoO_x_ and PMMA films as estimated by cyclic voltammetry measurements. The reference electrode was Ag/AgCl. Table S1: Quantitive analysis, weight and atomic ratios in percentage for oxygen (O, K series), molybdenum (Mo, L series), and tungsten (W, L series) of W-doped MoO_x_ sample deposited in N_2_ environment.
