# Supplementary material for: Flexible Inorganic/Organic Memristor Based on W-Doped MoOx/Poly(methyl methacrylate) Heterostructure
Source: Nanomaterials (Basel). 2025 Nov 12;15(22):1707. doi: 10.3390/nano15221707 (PMC12655515; doi:10.3390/nano15221707)
Supplement: Supplementary file 1 [file nanomaterials-15-01707-s001.zip › nanomaterials-3805053-supplementary.pdf]

Flexible Inorganic/Organic Memristor Based on W-Doped MoO_x/Poly(Methyl Methacrylate) Heterostructure

Gion Kalemai [1], Konstantinos Aidinis [2,3], Elias Sakellis [4,5], Petros-Panagis Filippatos [4], Polychronis Tsipas [1], Dimitris Davazoglou [4] and Anastasia Soultati [4,*]

[1] Department of Physics, University of Patras, Rio, 26504 Patra, Greece; c.kalemai@iit.demokritos.gr (G.K.)
[2] Department of Electrical and Computer Engineering, Ajman University, Ajman P.O. Box 346, United Arab Emirates
[3] Center of Medical and Bio-Allied Health Sciences Research, Ajman, United Arab Emirates
[4] Institute of Nanoscience and Nanotechnology (INN), National Center for Scientific Research Demokritos, Agia Paraskevi, 15341 Athens, Greece; e.sakellis@inn.demokritos.gr (E.S.); petpanfilippatos@gmail.com (P.-P.F.)
[5] Solid State Physics Section, Department of Physics, National and Kapodistrian University of Athens, Panepistimioupolis, Zografos, 15784 Athens, Greece
* Correspondence: a.soultati@inn.demokritos.gr

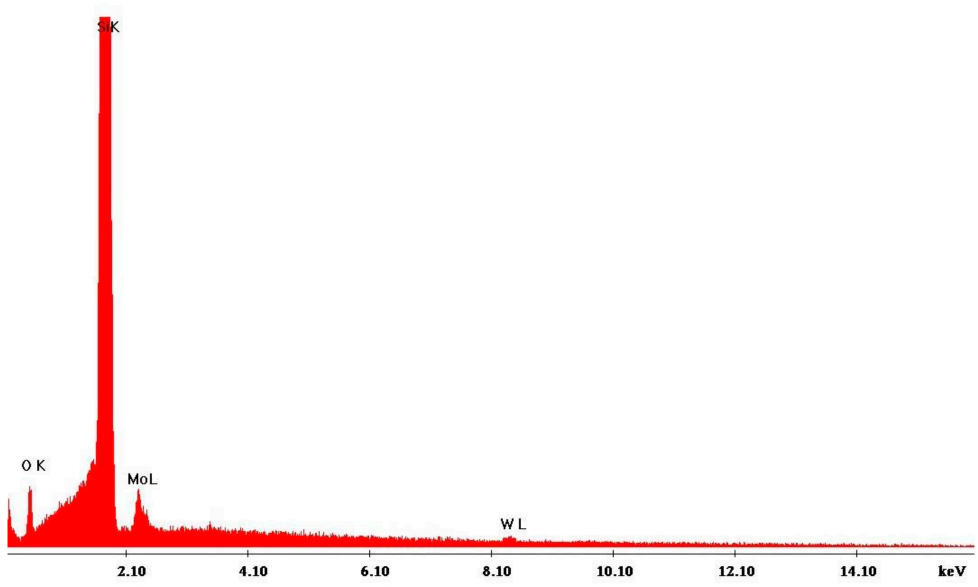

Figure S1: EDS image of W-doped MoO_x film deposited on silicon substrate in N2 environment.

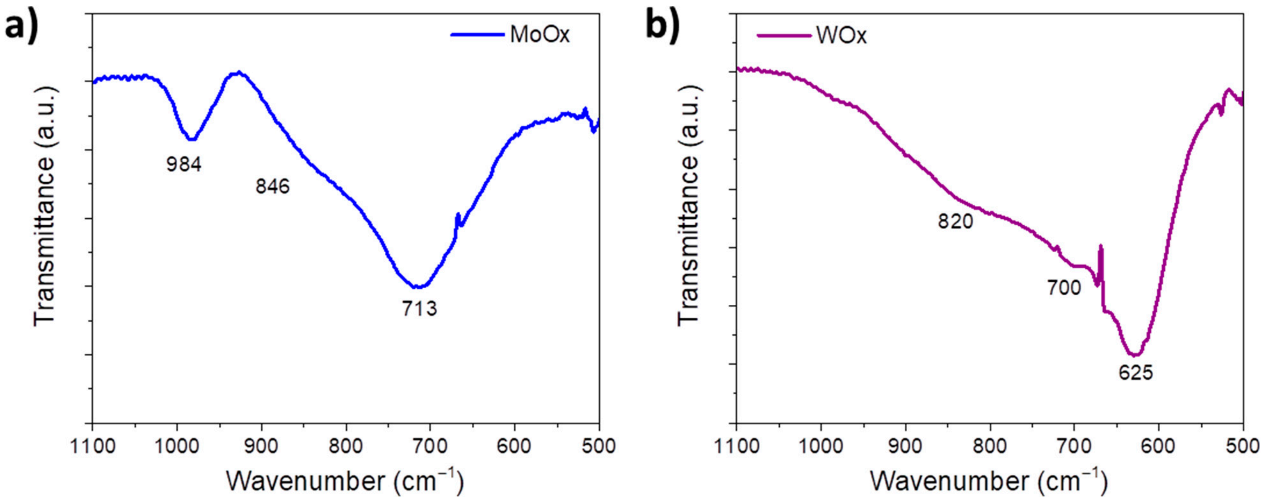

Figure S2: FTIR transmittance spectra of a) molybdenum and b) tungsten oxide thin films.

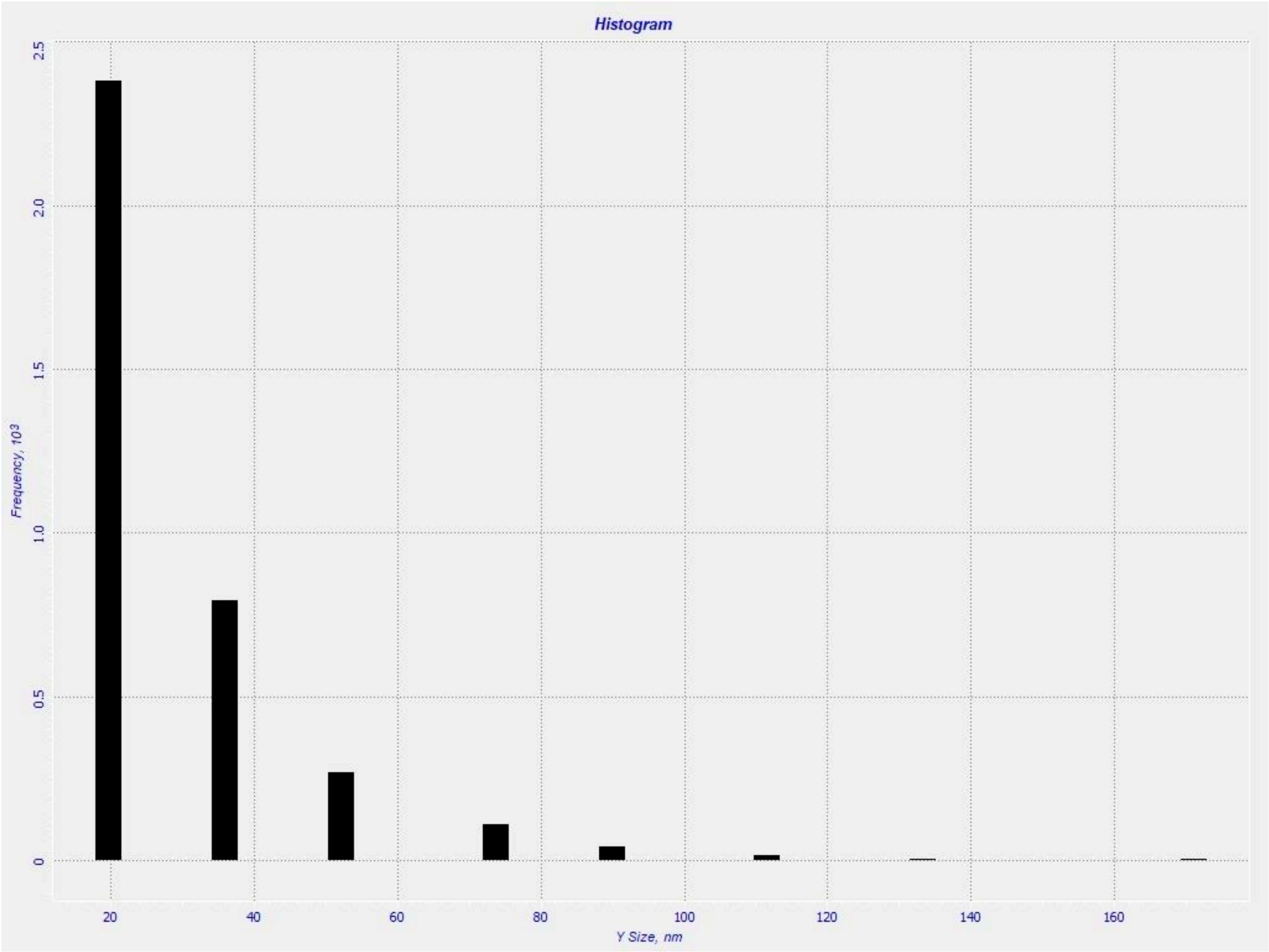

Figure S3: Grain analysis of 5x5 μm^2 AFM image of W-MoO_x surface.

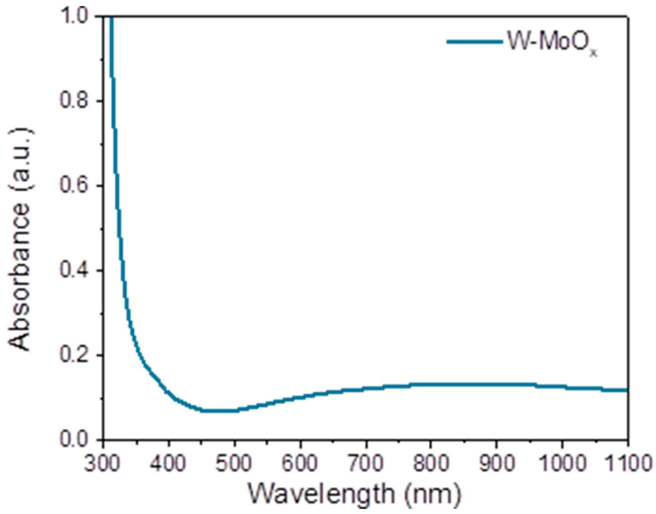

Figure S4: Absorbance spectrum of the mixed W-MoO$_x$ film deposited on glass substrate.

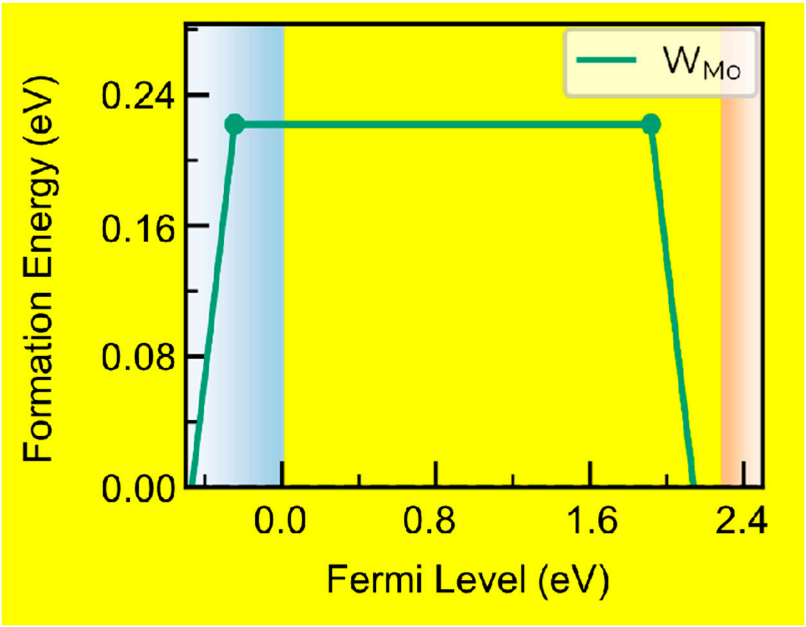

Figure S5: The formation energy versus the fermi level for the O-rich conditions using r^2SCAN+D3 functional.

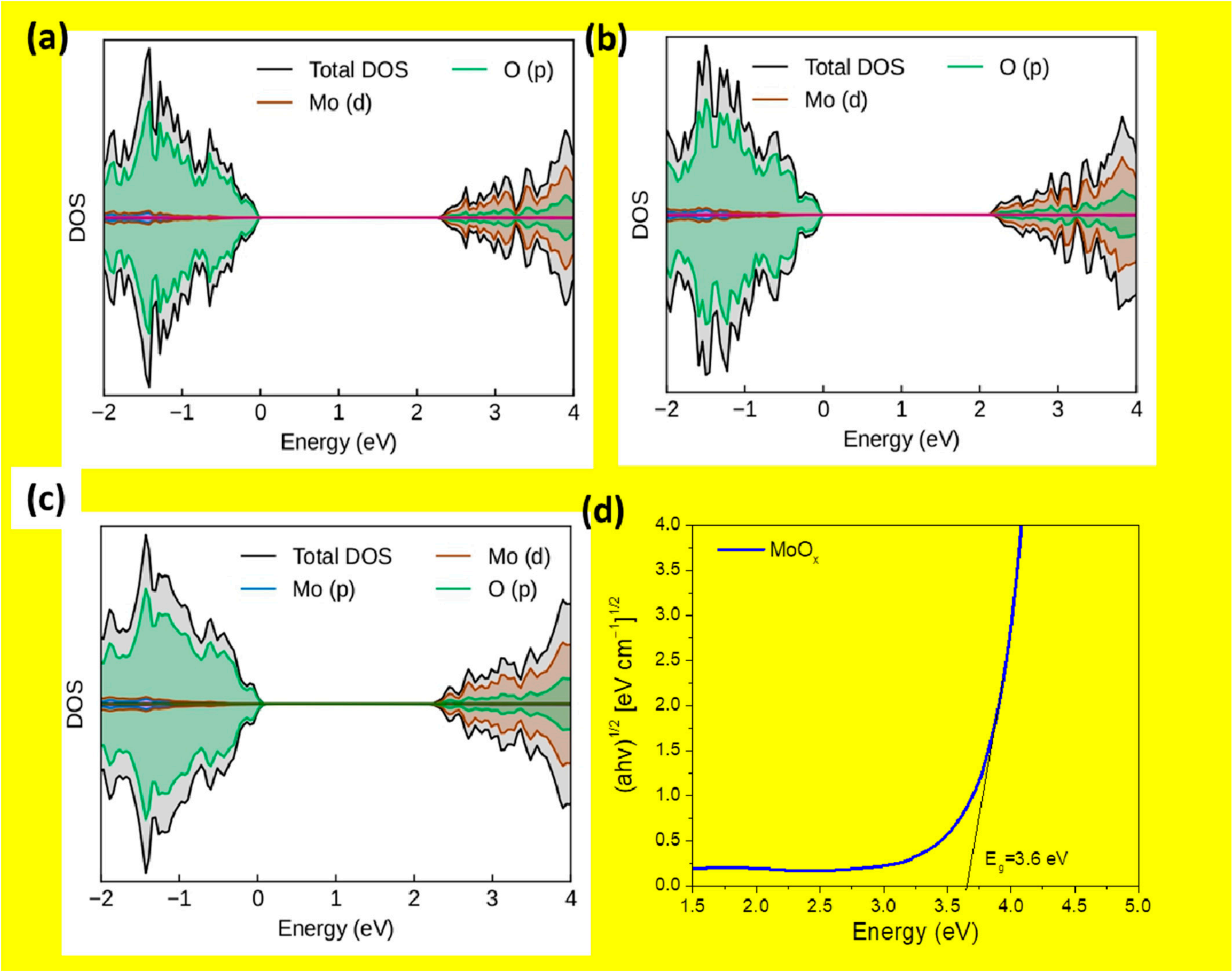

Figure S6: The density of states plot for the a) neutral and b) -1 charge W_{MO}^{0}:MoO_3 and c) undoped MoO .

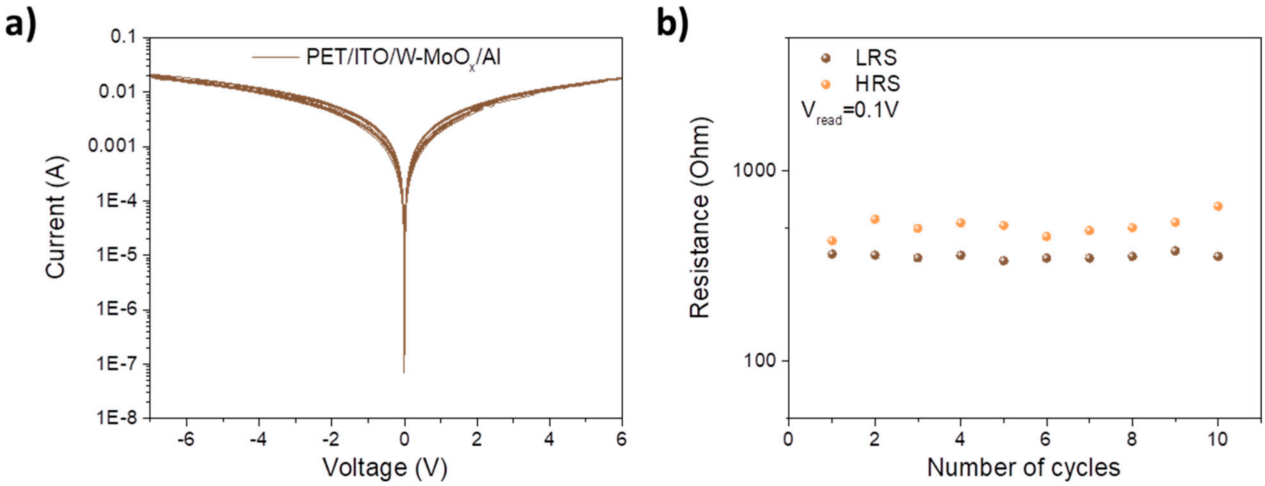

Figure S7: I-V characteristic curves of the PET/ITO/W-MoO_x/Al memristor without the PMMA film.

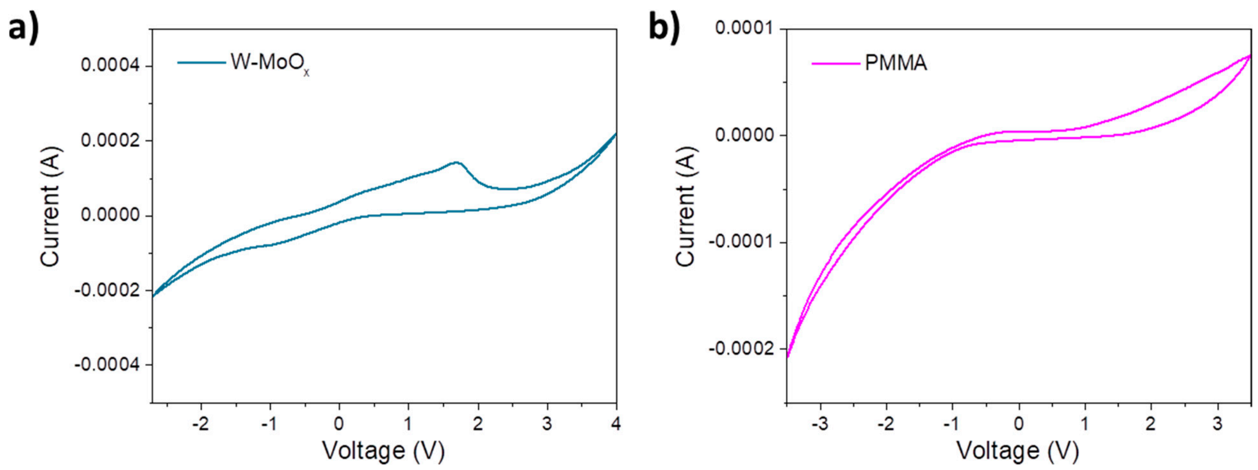

Figure S8: Cyclic voltammetry measurements of a) W-MoO$_x$ and b) PMMA films coated on ITO substrates.

Note S1: Energy levels of W-MoO$_x$ and PMMA films as estimated by cyclic voltammetry measurements. The reference electrode was Ag/AgCl.

The reduction potential of W-MoO$_x$ versus Ag/AgCl is E_{ox}= - 1.02 V

The oxidation potential of Fc/Fc$^+$ is: 0.62 V

The reduction potential of W-MoO$_x$ versus Fc/Fc$^+$ is: 0.4 V

The resulting conduction band (CB) of W-MoO$_x$ versus vacuum level is: CB = - (4.8 -0.4) = -4.4 eV

The estimated valence band (VB) of W-MoOx versus vacuum level is: VB = -(CB + E_g) = -(4.4+3.4) = -7.8 eV

The reduction potential of PMMA versus Ag/AgCl is: E_{red} = - 1.26 V

The oxidation potential of Fc/Fc$^+$ is: 0.62 V

The reduction potential of PMMA versus Fc/Fc$^+$ is: $E_{red}^{Fc/Fc+}$ = - 1.26 – 0.62 = -1.88 V

The resulting in the estimated LUMO energy level versus vacuum: E_{LUMO} = -(4.8 – 1.88) eV = -2.92 eV

The estimated HOMO of PMMA versus vacuum level is: HOMO = -(LUMO + E_g) = -(2.92+5.0[1]) = -7.92 eV

Table S1: Quantitive analysis, weight and atomic ratios in percentage for oxygen (O, K series), molybdenum (Mo, L series), and tungsten (W, L series) of W-doped MoO_x sample deposited in N_2 enviroment.

Element	Weight ratio (%)	Atomic ratio (%)
O (K series)	38.94	83.10
Mo (L series)	32.69	11.63
W (L series)	28.37	5.27
Total	100	100

References

1. Ayesh, A.S. Electrical and optical characterization of PMMA doped with $Y_{0.0025}Si_{0.025}Ba_{0.9725}$ $(Ti_{(0.9)}Sn_{0.1})O_3$ ceramic. *Chin J Polym Sci* **2010**, *28*, 537–546.
